# Investigation on Surface Polarization of Al_2_O_3_-capped GaN/AlGaN/GaN Heterostructure by Angle-Resolved X-ray Photoelectron Spectroscopy

**DOI:** 10.1186/s11671-017-2271-x

**Published:** 2017-08-17

**Authors:** Tian Li Duan, Ji Sheng Pan, Ning Wang, Kai Cheng, Hong Yu Yu

**Affiliations:** 1 Southern University of Science and Technology, Shenzhen, 518055 People’s Republic of China; 2 Institute of Materials Research and Engineering, A*STAR (Agency for Science, Technology and Research), 2 Fusionopolis Way, Innovis, #08-03, Singapore, 138634 Singapore; 3 Enkris Semiconductor, Inc, Suzhou, 215000 People’s Republic of China

**Keywords:** Surface polarization, Interfacial layer, ARXPS, GaN heterostructure

## Abstract

The surface polarization of Ga-face gallium nitride (GaN) (2 nm)/AlGaN (22 nm)/GaN channel (150 nm)/buffer/Si with Al_2_O_3_ capping layer is investigated by angle-resolved X-ray photoelectron spectroscopy (ARXPS). It is found that the energy band varies from upward bending to downward bending in the interface region, which is believed to be corresponding to the polarization variation. An interfacial layer is formed between top GaN and Al_2_O_3_ due to the occurrence of Ga–N bond break and Ga–O bond forming during Al_2_O_3_ deposition via the atomic layer deposition (ALD). This interfacial layer is believed to eliminate the GaN polarization, thus reducing the polarization-induced negative charges. Furthermore, this interfacial layer plays a key role for the introduction of the positive charges which lead the energy band downward. Finally, a N_2_ annealing at 400 °C is observed to enhance the interfacial layer growth thus increasing the density of positive charges.

## Background

Gallium nitride (GaN) is considered as one of the most attractive semiconductor materials in many fields ranging from LED industries to power electronic industries [1, 2]. The popularity is due to a number of advantages with respect to silicon: high breakdown electrical field, high electron mobility, and excellent thermal stability [3, 4]. GaN high-electron-mobility transistor (HEMT) is widely studied for high-power and high-frequency application [1, 5, 6]. In the HEMT, if the Schottky gate is adopted, this gate interface brings in large interface states which exacerbate the large leakage current and low breakdown field [7]. An insulator induced as the surface passivation layer and gate dielectric could help mitigate the above issues [8–10].

Al_2_O_3_ is preferred for such an insulator application due to its large band gap, high dielectric constant, and more negative Gibbs free energy comparing to Ga_2_O_3_, so it is believed that Al_2_O_3_ could passivate the surface states and improve the electric breakdown field [5]. However, an interfacial layer is formed inevitably at the GaN/Al_2_O_3_ interface after the deposition of the Al_2_O_3_ [11, 12]. This interfacial layer is believed to be correlated with reliability of the threshold voltage and property of two-dimensional electron gas (2DEG) and plays a key role to control the band bending [2, 13–16].

Although the interfacial layer has been studied by several research groups, the role played by the interfacial layer has not been exploited in depth [12, 17]. Therefore, in this paper, we use the angle-resolved X-ray photoelectron spectroscopy (ARXPS) to detect the gradual changes of the band bending and obtain the atomic structure of the interfacial layer [11]. Different thicknesses of Al_2_O_3_ are deposited on GaN samples by atomic layer deposition (ALD). ALD takes advantage of a low-temperature layer-by-layer deposition technology, which limits the thermal reaction between GaN and Al_2_O_3_. Thus, ALD is favored by the industries to deposit high-k dielectrics due to its high conformability and uniformity, accuracy on thickness control, high film quality, and low defect density [4]. This enables a smooth and low-defect Al_2_O_3_/GaN interface. After Al_2_O_3_ deposition, a 400 °C post-deposition annealing (PDA) sample is also prepared to intensify the interfacial layer reaction, enhancing the interfacial layer formation. Based on the ARXPS results, it is found that the band is bending upward initially from the GaN substrate to the near interface due to the polarization-induced negative charges. However, as an increase in detection angle *θ*, the band bends downward gradually because of the formation of positive charges [5, 11–13].

## Methods

The Ga-face GaN/AlGaN/GaN-on-Si(111) wafer was purchased from a commercial company (Enkris.com). The epitaxial wurtzite structure comprises a 2-nm GaN layer on top of a 22-nm AlGaN layer, and the two epitaxial layers are grown on a 150-nm i-GaN layer. A buffer layer serves as a transition layer connecting the GaN epilayer and the Si substrate. Three samples, S1, S2, and S3, are prepared. All samples were first decreased for 5 min in acetone, followed by immersion in isopropyl alcohol and a rinse in flowing deionized (DI) water. The native oxide was then etched away by dipping it into a dilute HCl solution (HCl:H_2_O = 1:10) for ~ 1 min, followed by a rinse in DI water. The Al_2_O_3_ are deposited by ALD on top of GaN, with trimethyl aluminum (TMA) and H_2_O as the metal precursor and oxidant, respectively. The Al_2_O_3_ thickness is 1 nm for sample S1 and 3 nm for samples S2 and S3. The thickness is measured by the ellipsometer. Moreover, S3 was subjected to PDA in N_2_ at 400 °C for 5 min.

ARXPS measurements were carried out in a Thermo Fisher Scientific Theta Probe system equipped with a monochromatic, microfocused Al Kα (1486.6 eV) X-ray source and a hemispherical electron energy analyzer. The binding energy (BE) calibration was performed using pure Ni, Au, Ag, and Cu standard samples by setting the Ni Fermi edge, Au 4*f*_7/2_, Ag 3*d*_5/2_, and Cu 2*p*_3/2_ peaks at 0.00 ± 0.02, 83.98 ± 0.02, 368.26 ± 0.02, and 932.67 ± 0.02 eV, respectively. The FWHM of a given component spectrum was allowed to vary within a narrow range only (± 0.1 eV). The lowest number of component spectra was used to obtain acceptably low residual values [11]. The XPS spectra were recorded at different detection angles (*θ*), ranging from 27.5° to 72.5° with respect to the sample normal in parallel without tilting the sample. To remove for possible positive charging-induced BE shift, the XPS spectra obtained were referenced to the C 1*s* peak at 285.0 eV. Quantitative analysis, including element/bond ratio determination, was achieved using relative sensitivity factors and algorithms embedded in the Avantage software [11].

## Results and discussion

**Fig. 1:**
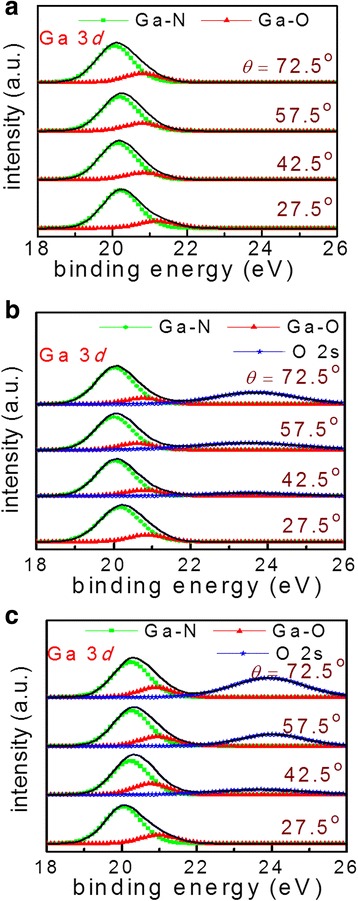
The XPS Ga 3*d* core-level spectra for **a** S1, **b** S2, and **c** S3

**Fig. 2:**
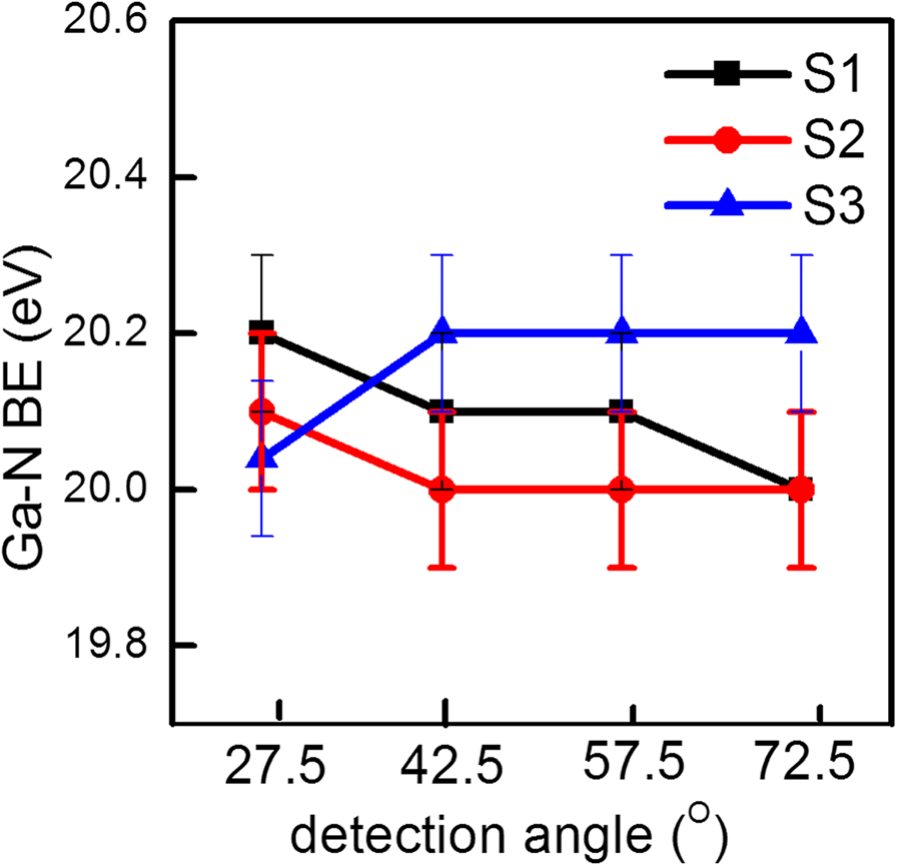
The BE of the Ga–N spectrum peak as a function of the detection angle *θ* (relative to the normal) for S2. The error bar is ± 0.1 eV

**Fig. 3:**
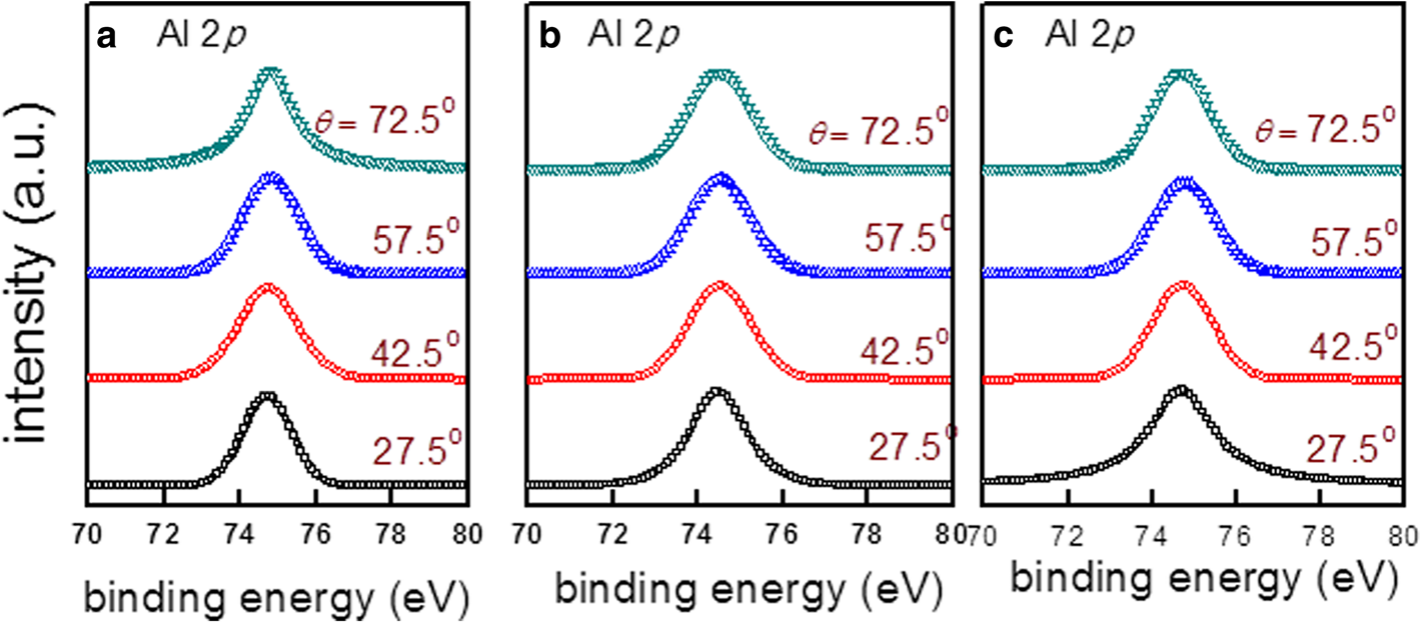
The XPS Al 2*p* core-level spectra for **a** S1, **b** S2, and **c** S3, and the peak indicates the Al–O bond. Moreover, there is no significant BE variation

The Ga 3*d* core-level spectra for S1–S3 at different photoelectron detection angles are depicted in Fig. 1a–c, respectively. For S1, each Ga 3*d* spectrum can be fitted with two peaks, corresponding to the Ga–N and Ga–O bonds. The Ga–O bonds are due to the oxide formation as a result of the oxygenant exposure in the ALD, and the oxygenant penetrated into the initial thin Al_2_O_3_ layer [3]. For S2 and S3, three peaks can be identified, noted as Ga–N, Ga–O, and O 2*s*, respectively. The O 2*s* peak is attributed to the Ga–O and Al–O bonds, and it becomes obvious when the detection angle is becoming larger. As this article is not focused on this peak, it will not be discussed further. Figure 2 presents the BE of Ga–N peaks as a function of *θ* for S1–S3. A 0.2-eV decrease is obtained from *θ* = 27.5° to 72.5° for S1. It suggests an upward band bending, which is consistent with the publications [3, 11]. For S2, the BE presents a decrease of 0.1 eV, indicating a mild upward band bending near the interface comparing to S1 or a flat energy band without band bending in consideration of experimental error. However, for S3, there is a 0.2-eV increase in the BE, which is in contrast to samples S1 and S2, advising a downward band bending. Figure 3 records the Al 2*p* spectra for all the samples, and there is no change in the BEs. Moreover, the peak is noted as the Al–O bond, and consequently, the AlGaN layer has negligible influence on the Ga–N BE variation. Table 1 summarizes the BEs of Ga 3*d* and Al 2*p* at different detection angles for all the samples, with the error of ± 0.1 eV.

**Table 1:** Summary of the BEs (eV) of Ga 3*d* and Al 2*p* at different detection angles for all the samples, with the error of ± 0.1 eV

Samples	Core level	Chemical bonds	Detection angles *θ*			
			27.5°	42.5°	57.5°	72.5°
S1	Ga 3*d*	Ga–N	20.2	20.1	20.1	20.0
		Ga–O	21.2	20.8	20.8	20.8
	Al 2*p*	Al–O	74.7	74.7	74.7	74.7
S2	Ga 3*d*	Ga–N	20.1	20.0	20	20.0
		Ga–O	20.8	20.8	20.6	20.7
	Al 2*p*	Al–O	74.6	74.7	74.7	74.7
S3	Ga 3*d*	Ga–N	20.0	20.2	20.2	20.2
		Ga–O	20.9	20.8	20.9	20.9
	Al 2*p*	Al–O	74.7	74.7	74.7	74.6

The ratios of the Ga–O to Ga–N peak for all the samples are given in Table 2. The ratio is around 0.2 for samples S1 and S2, which is consistent with previous results [3]. However, after the PDA treatment, the ratio increases to ~ 0.3 and denotes an increase of the GaO_*x*_ interfacial layer. Moreover, the Ga/N ratio is also given in Table 2. The ratio is calculated by comparing the integrated intensities of the Ga 3*d* and N 1*s* peaks with atomic sensitivity factors [18]. For samples S1 and S2, the ratio around 1.7 suggests a Ga-rich interfacial layer. However, after the N_2_ annealing, the ratio decreases to ~ 1.0. Furthermore, the sampling depth is also given in Table 2 for each angle. Because of the exponential attenuation of photoelectrons, 63 and 95% of detected electrons originate from within a distance of 1λ (i.e., λ represents electron’s inelastic mean free path (IMFP)) and 3λ, respectively, of the surface. Therefore, the XPS sampling depth is defined as 3λ nanometers underneath the sample surface. In our case, Al_2_O_3_ is the capping layer and the λ of Ga 3*d* photoelectrons in Al_2_O_3_ is estimated as ~ 3.4 nm. For a rough estimation, the sampling depth at different angles is given as 3λcos*θ*. However, the actual Ga–N BE sampling depth should consider the thickness of Al_2_O_3_, so the sampling depth is estimated as 3λcos*θ* minus the capping Al_2_O_3_ thickness. Because the GaO_*x*_ layer is on top of GaN heterostructure, the signal of this layer is included for every detection angle. However, with the increase of the detection angle, the signal intensity of the Ga–N bond is decreased, resulting in the increase of the Ga–O/Ga–N ratio. Comparing S2 to S3, the increase of the Ga–O/Ga–N ratio and the decrease of the Ga/N ratio suggest the Ga-rich layer has been oxidized to form GaO_*x*_.

**Table 2:** Summary of the peak intensity ratio of Ga–O to Ga–N, Ga to N, and the corresponding XPS sampling depth at different detection angles *θ*

Samples	Al_2_O_3_ thickness (nm)	Ratio	Detection angles *θ*			
			27.5°	42.5°	57.5°	72.5°
S1	1	Ga–O/Ga–N	0.19	0.19	0.23	0.25
		Ga/N	2.03	1.69	1.60	1.59
		Ga–N BE sampling depth (nm)	8.0	6.5	4.5	2.1
S2	3	Ga–O/Ga–N	0.19	0.17	0.2	0.16
		Ga/N	1.92	1.72	1.85	1.69
		Ga–N BE sampling depth (nm)	6.0	4.5	2.5	0.1
S3	3	Ga–O/Ga–N	0.23	0.33	0.27	0.28
		Ga/N	0.94	1.15	1.39	1.09
		Ga–N BE sampling depth (nm)	6.0	4.5	2.5	0.1

Sampling depth = 3λcos*θ*—the capping Al_2_O_3_ thickness

**Fig. 4:**
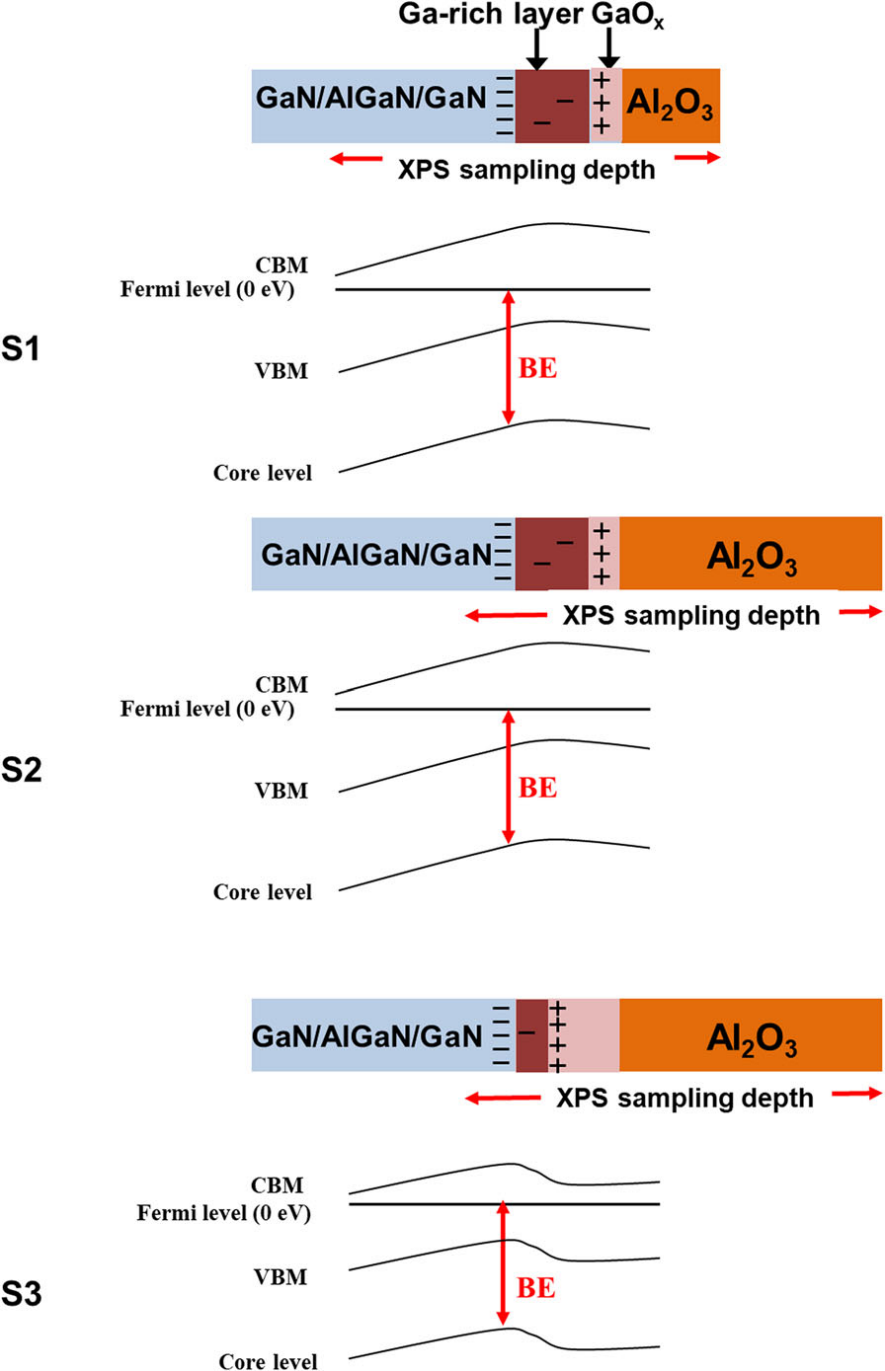
In the interface region, O replacing N in the Ga–N bond results in a Ga-rich layer and a GaO_*x*_ layer. The Ga-rich layer acts as the GaN-to-GaO_*x*_ transition layer. The Ga–O formation eliminates the polarization of GaN and acts as positive charges. As a result, the conduction band bends gradually from upward to downward and the BE varies accordingly

To illustrate the experiment data presented here, a model is schematically proposed in Fig. 4. The Fermi energy level of the GaN/AlGaN/GaN substrate is calibrated as 0 eV during XPS measurement [19]. The conduction band maximum (CBM), valence band maximum (VBM), and core level are given. The BE is the energy difference between the core level and Fermi level. In the ALD deposition, O from the oxygenant could replace N in the Ga–N bond to oxidize the GaN and the surrounding N atoms could form N_2_ molecules [20], which leads to the formation of the Ga-rich layer and the gallium oxide (GaO_*x*_) interfacial layer [11, 18]. This is supported by the Ga/N ratio which is larger than 1 in Table 2. This ratio indicates the change of GaN stoichiometry, and the intrinsic spontaneous polarization effect of GaN should disappear [21–23]. As a result, the Ga-rich layer, as the GaN-to-GaO_*x*_ transition layer, eliminates the polarization-induced negative charges and results in a flat conduction band [11], as shown in Fig. 4.

Furthermore, in the annealing process, the Ga-rich surface is oxidized to form a thicker GaO_*x*_ layer. Because the oxidation is a kinetically limited reaction and is restricted to about two surface monolayers, the bulk will not be strongly perturbed [24]. Therefore, the Ga–N bond signal is mainly from the unoxidized underlying bulk, resulting in the Ga/N stoichiometric ratio of 1 for S3. The GaO_*x*_ layer has been reported to bring in positive charges which may be interfacial fixed charges with energy states between the conduction band minima of the native oxide and GaN, which would bend the band downward [4, 11, 13, 14]. Therefore, the conduction band of the Ga-rich layer starts to decrease in the region near the GaO_*x*_ layer. A thicker GaO_*x*_ is expected to bring in larger density of positive charges. With respect to the constant BE of Ga–O and Al–O in S3, it indicates that the positive charge should locate at the interface of the Ga-rich layer/GaO_*x*_ layer. The positive charges and polarization-induced negative charges build an internal electric filed which modified the band bending from upward band bending to downward band bending, shown in Fig. 4. Because of the downward band bending, the BE increases with the increase of detection angle.

The GaO_*x*_ interfacial layer brings in positive charges which increase interface barrier height ɸ_b_. ɸ_b_ is defined as the energy difference between the Fermi level and the conduction band minimum at the surface or interface [25]. As a result, after the A_2_O_3_ deposition, the mobility of the 2DEG is increased and the electron density of 2DEG is decreased [16, 25, 26].

With the increase of the Al_2_O_3_ thickness, the XPS signal reflects more on the interface region between the capped Al_2_O_3_ and GaN/AlGaN/GaN, which is validated by the XPS sampling depth shown in Table 2. This explains that only part of the band bending profile could be detected for S2 [27]. As a result, the BE variation is 0.1 eV, smaller than 0.2 eV of S1. For S3, with a thicker interfacial layer, the density of positive charges is increased resulting in a downward band bending.

## Conclusions

In summary, the interface polarization of Al_2_O_3_-capped GaN/AlGaN/GaN is investigated by the ARXPS. The intrinsic polarization of GaN is eliminated because of a Ga-rich layer and a GaO_*x*_ layer formation. Moreover, the Ga–O bonds from the GaO_*x*_ layer bring in positive interfacial fixed charges. Due to this polarization variation, the band varies from the upward bending to the downward bending in the interface region.
